# Non-contact electrical stimulation via a Vector-potential transformer promotes bone healing in drill-hole injury model

**DOI:** 10.1007/s00774-025-01603-0

**Published:** 2025-04-29

**Authors:** Nao Yashima, Wataru Minamizono, Hiroya Matsunaga, Jiazheng Lyu, Kaoru Fujikawa, Hirai Suito, Takumi Okunuki, Shingo Nakai, Masafumi Ohsako

**Affiliations:** 1 Graduate School of Health and Sports Science, Toyo University, 1-7-11 Akabanedai, Kita-ku, Tokyo, 115-8650 Japan; 2 Graduate School of Human Life Design, Toyo University, 1-7-11 Akabanedai, Kita-ku, Tokyo, 115-8650 Japan; 3 Department of Oral Anatomy, Showa Medical University School of Dentistry, 1-5-8 Hatanodai, Shinagawa-ku, Tokyo, 142-0064 Japan; 4 Department of Anatomy, Teikyo University School of Medicine, 2-11-1 Kaga, Itabashi-ku, Tokyo, 173-8605 Japan; 5 Japan Society for the Promotion of Science, Research Organization of Science and Technology, Ritsumeikan University, 5-3-1 Kojimachi, Chiyoda-ku, Tokyo, 102-0083 Japan; 6 Department of Judo Seifuku and Health Sciences, Tokoha University School of Health Promotional Sciences, 1230 Miyakoda-cho, Hamana-ku, Hamamatsu-shi, Shizuoka, 431-2102 Japan; 7 Department of Health and Sports Science, Toyo University School of Health and Sports Science, 1-7-11 Akabanedai, Kita-ku, Tokyo, 115-8650 Japan

**Keywords:** Vector-potential transformer, Non-contact electrical stimulation, Bone healing, Drill-hole injury model, Rat

## Abstract

**Introduction:**

We investigated the effects of non-contact electrical stimulation via a Vector-potential (VP) transformer, a novel physical therapy device, on bone healing in drill-hole injury models.

**Materials and methods:**

Six-week-old male Wistar rats, after a one-week acclimation period, were divided into three groups: the control group (CO), the bone injury group (BI), in which a drill-hole injury was created, and the VP stimulation group (VP), which received non-contact electrical stimulation via a VP transformer after bone injury. In the VP group, rats underwent stimulation at 200 kHz for 30 minutes per day, seven days per week.

**Results:**

The VP group exhibited increased bone formation as early as day 7 post-injury, with significantly higher bone volume than the BI group at all time points (day 7: p = 0.0003; day 14: p = 0.0024; day 21: p = 0.0001). By day 21, the VP group showed lighter toluidine blue staining and reduced biglycan immunoreactivity compared to the BI group. Bone mineral density also increased (p = 0.0008). Osteoblasts in the VP group displayed abundant cytoplasm and a high capacity for osteocalcin synthesis. Additionally, the VP group demonstrated increased expression of *Bglap* (day 5: p = 0.0068; day 7: p = 0.0096) and *Ctsk* (day 7: p = 0.0329; day 14: p = 0.0171), along with a higher number of TRAP-positive osteoclasts (day 21: p = 0.0159) compared to the BI group.

**Conclusion:**

Non-contact electrical stimulation via a VP transformer promotes bone healing in drill-hole injury models.

## Introduction

As the global population continues to age, the incidence of fractures is rising dramatically [1]. While most fractures heal naturally over time [2], complications such as delayed healing or non-union occur in 5–10% of cases, often due to aging or chronic conditions such as osteoporosis and diabetes [3–6]. Hip and spine fractures, which are particularly prevalent among individuals with osteoporosis [7, 8], not only reduce quality of life but also significantly shorten life expectancy [9, 10]. Additionally, the global economic burden of fracture-related healthcare costs is increasing, placing substantial strain on healthcare systems [11]. Given these challenges, accelerating fracture healing is of great significance.

Various physical therapies, including electrical stimulation [12], low-intensity pulsed ultrasound [13], and extracorporeal shockwave therapy [14], have shown promise in promoting fracture healing. However, these treatments typically require direct contact with the skin via probes or electrode pads, which may necessitate cast removal and impose additional discomfort or inconvenience on patients [15, 16]. Pulsed electromagnetic field (PEMF) therapy has gained attention as a non-contact alternative [17]. Clinical studies have demonstrated its effectiveness in enhancing fracture healing [18, 19], making it an attractive option for both patients and healthcare providers. However, despite its potential, PEMF therapy often requires prolonged use, which can lead to patient fatigue and financial strain [20, 21].

In this context, a newly developed device known as the Vector-potential transformer has gained attention [22]. This device generates a Vector potential, which is a precursor to an electric field, within a cylindrical space by directing current through a solenoid coil. This mechanism enables non-contact electrical stimulation without the need for probes or electrode pads [22]. Leveraging this technology, we have previously investigated the effects of non-contact electrical stimulation delivered by the Vector-potential transformer, i.e., VP stimulation, on the musculoskeletal system in rats, revealing various biological effects on the bone and cartilage [23, 24]. These findings confirm that VP stimulation can influence tissues and cells without direct contact. Given these effects, VP stimulation may also promote fracture healing; however, this potential has not yet been fully explored. Investigating this possibility would be valuable in establishing the VP transformer as a novel therapeutic tool for fracture management.

In this study, we examined the effects of VP stimulation on bone healing in a drill-hole injury model using histological and biochemical analyses. Through this fundamental research, we aimed to elucidate the therapeutic potential of the VP transformer in fracture treatment.

## Materials and methods

This study was conducted in accordance with the ARRIVE 2.0 guidelines, specifically the ARRIVE Essential Checklist [25]. This study was approved by the Animal Experimentation Committee of Toyo University (Tokyo, Japan; Approval No. 2022-11). Throughout the study, strict hygiene management was maintained for both the experimenters and the laboratory to minimize the risk of infection.

### Study design

**Fig. 1:**
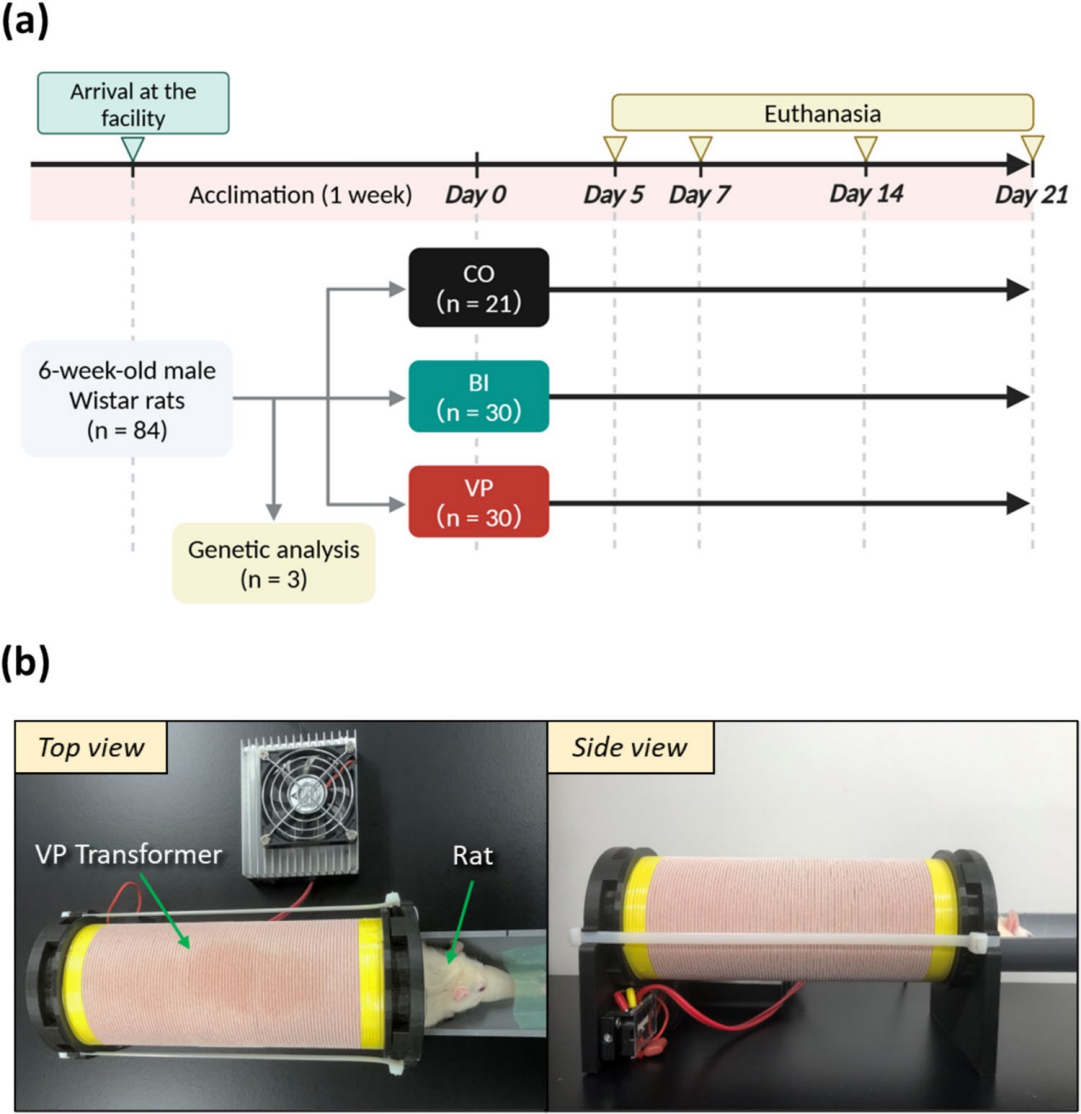
Study design. **a** Flowchart. *CO* Control,* BI*: Bone injury, *VP* Vector-Potential. Rats in each group were euthanized by CO₂ inhalation on days 5, 7, 14, and 21 after the start of the experiment. **b** VP transformer intervention. The hind limb of the anesthetized rat was fully inserted into the VP transformer for stimulation. *VP* Vector-potential

Six-week-old male Wistar rats (n = 84; body weight, 130–150 g; Nippon Bio-Supp. Center; Tokyo, Japan) were used as experimental animals. The rats were delivered as specific pathogen-free and housed under controlled conditions with a temperature of 24 ± 2 °C, humidity of 50 ± 5%, and a 12-h light/dark cycle, with a maximum of four rats per cage. The rats had free access to water and solid food (Oriental Yeast, Tokyo, Japan). After a 1-week acclimation period, the rats, excluding three used for genetic analysis (n = 3), were divided into three groups: the control group (CO, n = 21), the bone injury group (BI, n = 30), in which a drill-hole injury was created, and the group receiving non-contact electrical stimulation via a VP transformer after bone injury (VP, n = 30) (Fig. 1a). In the VP group, rats were anesthetized by isoflurane inhalation (flow rate: 1 L/min, induction concentration: 4%, maintenance concentration: 2%), and VP stimulation (200 kHz, 30 min/day, 7 days/week) was applied using a VP transformer (Sumida Inc., Miyagi, Japan) (Fig. 1b). All rats were euthanized by CO_2_ inhalation on days 5, 7, 14, and 21 after the experiment and tibial samples were collected (CO: n = 3 on day 5 and n = 6 on days 7, 14, and 21; BI and VP: n = 6 on day 5 and n = 8 on days 7, 14, and 21). The experimental results were shared among the eight co-authors under blinded conditions, and comprehensive interpretations were obtained.

### Preparation of drill-hole injury models

A standardized drill-hole injury model was created under direct visual observation to ensure surgical simplicity and experimental reproducibility, following previous studies [26]. For general anesthesia, medetomidine hydrochloride (0.375 mg/kg; 907220, Nippon Zenyaku Kogyo, Tokyo, Japan), midazolam (2 mg/kg; 614243022, Sandoz, Tokyo, Japan), and butorphanol tartrate (2.5 mg/kg; 88957, Meiji Animal Health, Tokyo, Japan) were administered via intraperitoneal injection. Once deep anesthesia was confirmed, the skin over the central tibia was shaved and incised, and the space between the gracilis muscle (proximal) and semitendinosus muscle (distal) was widened to expose the tibial cortical bone. A 2.0-mm diameter drill hole was then created in the upper-middle third of both tibiae using a drill bar (89365, Meisinger, Neuss, Germany). After drilling, the surrounding area was rinsed with saline, and the skin was sutured to complete the procedure. Postoperatively, anesthesia was reversed with atipamezole hydrochloride (0.75 mg/kg; 006030, Nippon Zenyaku Kogyo), and analgesia was provided via intraperitoneal injection of buprenorphine (0.3 mg/kg; 5L77L3, Otsuka Pharmaceutical, Tokyo, Japan).

### Micro-computed tomography (µCT) analysis

The extracted samples (n = 6) were scanned using µCT (Sky Scan 1276, Bruker, Kontich, Belgium). The scanning conditions were set at 70 kV voltage, 57 µA current, 360° full rotation, and 0.20° rotation step. The acquired scan data were analyzed using 3D image processing software integrated into the Sky Scan system.

**Fig. 2:**
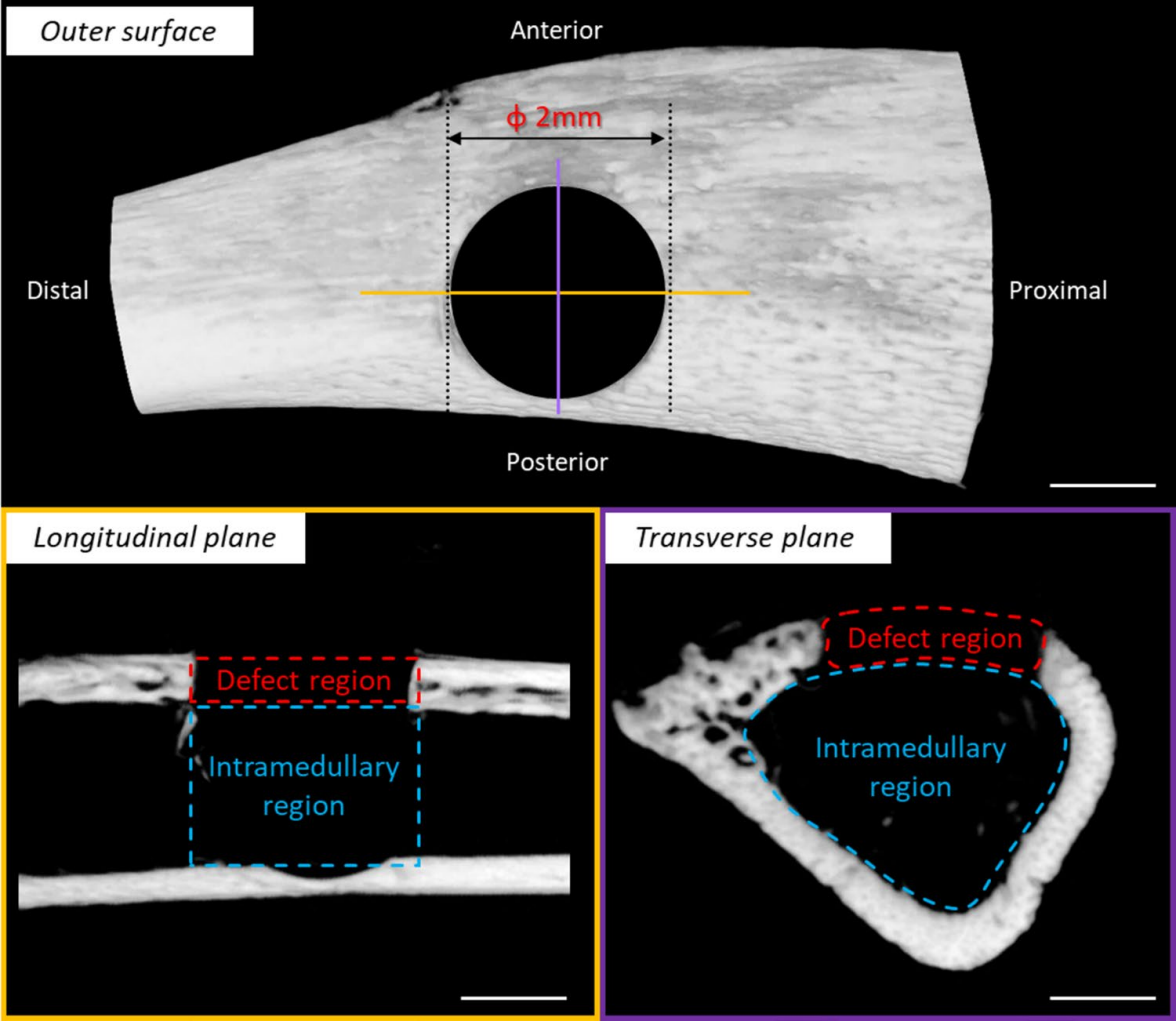
Overview of the drill-hole injury and its observation regions. The portion of the drill-hole injury corresponding to the height of the cortical bone was defined as the defect region, while the deeper portion at the level of the bone marrow cavity was defined as the intramedullary region. Scale bar = 1 mm

Based on a previous study [27], the drill-hole was divided into two distinct regions: the defect region, corresponding to the height of the existing cortical bone, and the intramedullary region, representing the deeper area at the level of the bone marrow cavity (Fig. 2). The bone volume (BV) and bone mineral density (BMD) of the new bone were assessed in the defect region, whereas BV was measured in the intramedullary region.

### Tissue processing

For macroscopic and histological analysis, the samples were allocated as follows: macroscopic observation (n = 2), resin-embedded polished sections (CO: n = 4; BI and VP: n = 5), and decalcified paraffin-embedded sections (n = 3).

The tibiae for macroscopic observation were treated with a 3% sodium hypochlorite solution (195-02206, Wako, Osaka, Japan) to remove soft tissue before observation.

The tibiae for resin-embedded polished sections were embedded in a mixed resin of Rigolac 2004 (3801, Nisshin EM, Tokyo, Japan) and Rigolac 70F (380, Nisshin EM). After embedding, the blocks were polished and sliced into longitudinal sections of approximately 150 µm thickness, followed by toluidine blue staining (209-14545, Wako).

The tibiae for decalcified paraffin-embedded sections were decalcified in 8% EDTA (348-01355, Dojindo, Tokyo, Japan) for 3 weeks, embedded in paraffin (166-18964, Wako), and sliced into longitudinal sections of approximately 4 µm thickness using a microtome.

### Hematoxylin and eosin (HE) staining, tartrate-resistant acid phosphatase (TRAP) staining

Decalcified paraffin sections were deparaffinized with letrozole and alcohol, followed by H&E staining (Mayer’s hematoxylin: 517-28-2, Sigma, Tokyo, Japan; eosin: E4382-25G, Sigma).

Additional sections were stained with TRAP staining solution prepared with naphthol AS-MX phosphate (N5000-1G, Sigma), Fast Red Violet LB Salt (F3381-500MG, Sigma), sodium hydrogen carbonate (191-01305, Wako), and L (+)-tartaric acid (207-00055, Wako) at 37 °C for 15 min. Methyl green (134-13901, Wako) was used as a counterstain for TRAP staining.

### Immunohistochemical analysis

Deparaffinized sections were blocked with a blocking reagent (PK-4001; Vector Laboratories, Burlingame, CA, USA) for 60 min to inhibit endogenous enzyme activity. The sections were then incubated overnight at 4 °C with primary antibodies against biglycan (×100; bs-7552R-TR, BIS, Massachusetts, USA) or osteocalcin (×100; OSTCLN-101AP, Invitrogen, Massachusetts, USA). Biglycan was detected using a secondary antibody (30 min) and ABC reagent (30 min) from the VECTASTAIN ABC Rabbit IgG Kit (PK-4001, Vector) and DAB (SK-4100, Vector) for visualization. Osteocalcin was detected using goat anti-rabbit IgG H&L (Alexa Fluor 488; ×200, ab150077, Abcam, Cambridge, UK), followed by mounting with DAPI (ab104139, Abcam).

### Gene expression analysis

The extracted samples (CO: n = 3; BI and VP: n = 6) were homogenized under sterile conditions, and total RNA was extracted using TRIzol reagent (15596018, Invitrogen). Total RNA was reverse-transcribed into cDNA using an iScript™ gDNA Clear cDNA Synthesis Kit (1725034, Bio-Rad, Tokyo, Japan). The cDNA was mixed with various TaqMan probes and analyzed using qRT-PCR. TaqMan probes were used for *Gapdh* (Rn01775763_g1, Invitrogen) as the housekeeping gene, and *Bglap* (encoding osteocalcin, Rn00566386_g1, Invitrogen) and *Ctsk* (encoding cathepsin K, Rn00580723_m1, Invitrogen) as target genes. Quantitative real-time polymerase chain reaction (qRT-PCR) analysis was performed using a CFX96 Touch Real-Time PCR Detection System (47153; Bio-Rad), and the Ct values for all samples were calculated. The relative expression levels were calculated using the ^ΔΔ^Ct method [28].

### Statistical analysis

All data were analyzed using IBM SPSS Statistics software ver. 29 (IBM Corp., Armonk, New York, USA). First, the Shapiro–Wilk test was performed to confirm whether the data from each group followed a normal distribution. Unpaired t-tests were performed to compare BI and VP groups. A one-way analysis of variance followed by Tukey’s test was used to compare multiple groups among the CO, BI, and VP groups. Statistical significance was defined as p < 0.05.

## Results

No deaths related to surgery or anesthesia were observed in this study. After recovery from anesthesia, rats in both the BI and VP groups regained normal gait and were able to feed independently. There were no signs of discomfort, such as abnormal vocalization, self-injury, or inflammation at the surgical site. Additionally, body weight did not differ significantly among the groups, with values of 291.8 ± 9.02 g in the CO group, 306.4 ± 10.59 g in the BI group, and 294.61 ± 12.44 g in the VP group.

### VP stimulation promotes the formation and calcification of new bone

**Fig. 3:**
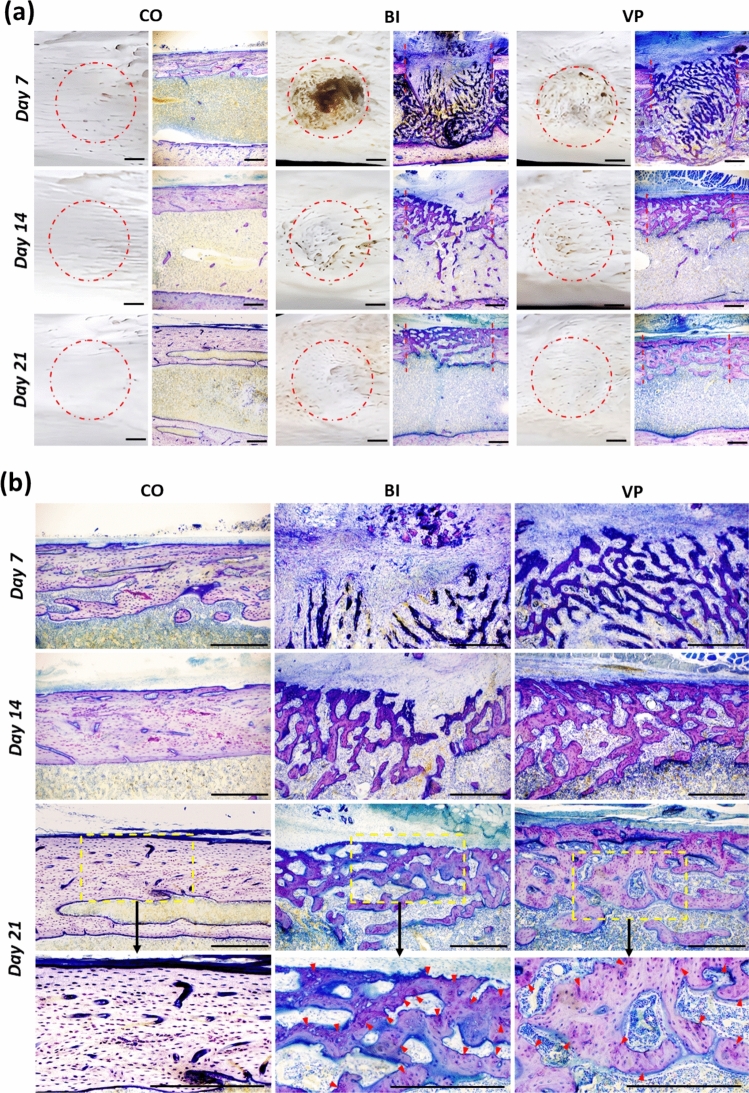
Representative histological images on days 7, 14, and 21 after drill-hole injury. **a** Appearance (left) and cross-section (right) of the drill-hole injury. The dotted line represents the edge of the drill-hole injury. Scale bar = 500 µm. **b** High magnification of new bone in the defect region. Red arrowheads indicate the metachromatic reaction observed in the bone matrix on day 21. Scale bar = 500 µm. *CO* Control; *BI* Bone injury; *VP* Vector-potential

Figure 3 illustrates the healing process of the drill-hole injury. Macroscopic observations revealed that in the BI group, new bone formation was observed on the surface of the drill-hole injury, extending from the edges toward the center by day 7; however, the drill-hole injury remained incompletely covered. In contrast, the VP group exhibited more extensive bone formation, with new bone visible not only at the edges but also at the center of the drill-hole injury. By days 14 and 21, the drill-hole injuries in both the BI and VP groups were completely filled with new bone; however, the VP group displayed a denser structure.

The longitudinal section analysis provided further insights. By day 7, the BI group exhibited thin, immature new bone that was intensely stained with toluidine blue, primarily in the intramedullary region oriented perpendicular to the long axis of the cortical bone. In contrast, the VP group showed new bone at the same time point with similar staining characteristics but was distributed across both the intramedullary and defect regions. Additionally, the new bone in the VP group was obliquely aligned parallel to the long axis of the cortical bone. This suggests that new bone formation in the VP group advanced further in bridging the drill-hole injury compared to that in the BI group. By days 14 and 21, new bone formation in the intramedullary region had decreased in both groups, with a more pronounced reduction in the VP group. On day 14, new bone with similar staining properties was present in the defect region in both groups; however, it was denser in the VP group. This difference became even more evident by day 21, as the VP group exhibited a reduced metachromatic reaction to a reddish-purple color compared to the BI group. In relation to this, the bone matrix in the VP group showed fainter staining than in the BI group, resembling the mature bone tissue observed in the CO group.

**Fig. 4:**
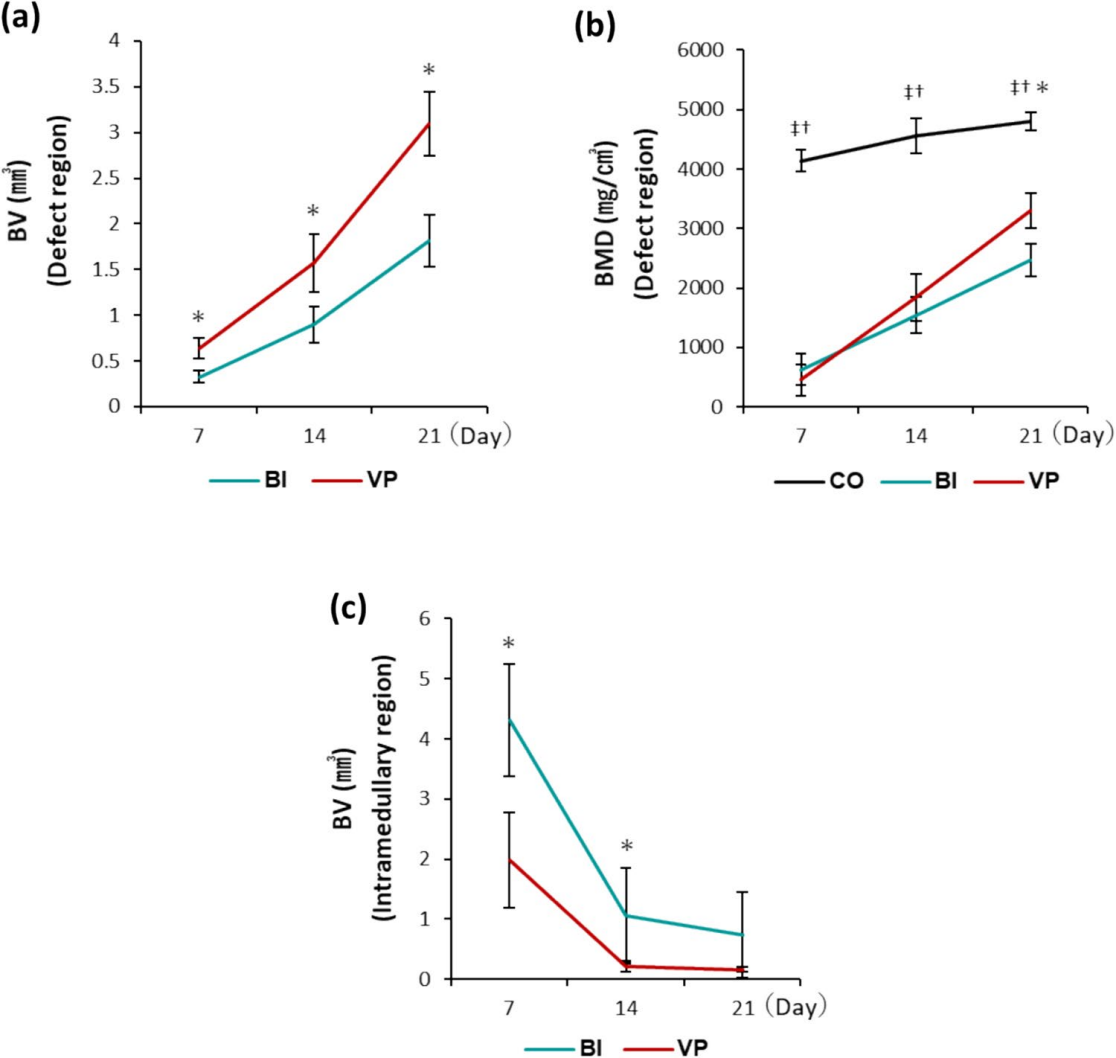
µCT analysis results of BV and BMD. **a** BV (defect region). ^*^p < 0.05 (between BI and VP). **b** BMD (defect region). ^‡^p < 0.05 (between CO and BI). ^†^p < 0.05 (between CO and VP). ^*^p < 0.05 (between BI and VP). **c** BV (intramedullary region). ^*^p < 0.05 (between BI and VP). **a−c** n = 6 per time point. *BV* bone volume, *BMD*, bone mineral density, *CO* Control, *BI*, Bone injury, *VP* Vector-potential, *µCT* micro-computed tomography

µCT analysis further confirmed these findings. BV in the defect region steadily increased in both groups, but the VP group consistently exhibited a significantly higher BV at all evaluation points (day 7: p = 0.0003; day 14: p = 0.0024; day 21: p = 0.0001; Fig. 4a). A similar trend was observed for BMD. While both groups showed a progressive increase, the VP group demonstrated higher BMD levels from day 14 onward, reaching statistical significance by day 21 (p = 0.0008; Fig. 4b). In contrast, BV in the intramedullary region progressively decreased over time in both groups; however, the VP group exhibited significantly lower values than the BI group on days 7 and 14 (day 7: p = 0.0008; day 14: p = 0.0281; Fig. 4c).

### VP stimulation reduces biglycan in the new bone matrix

**Fig. 5:**
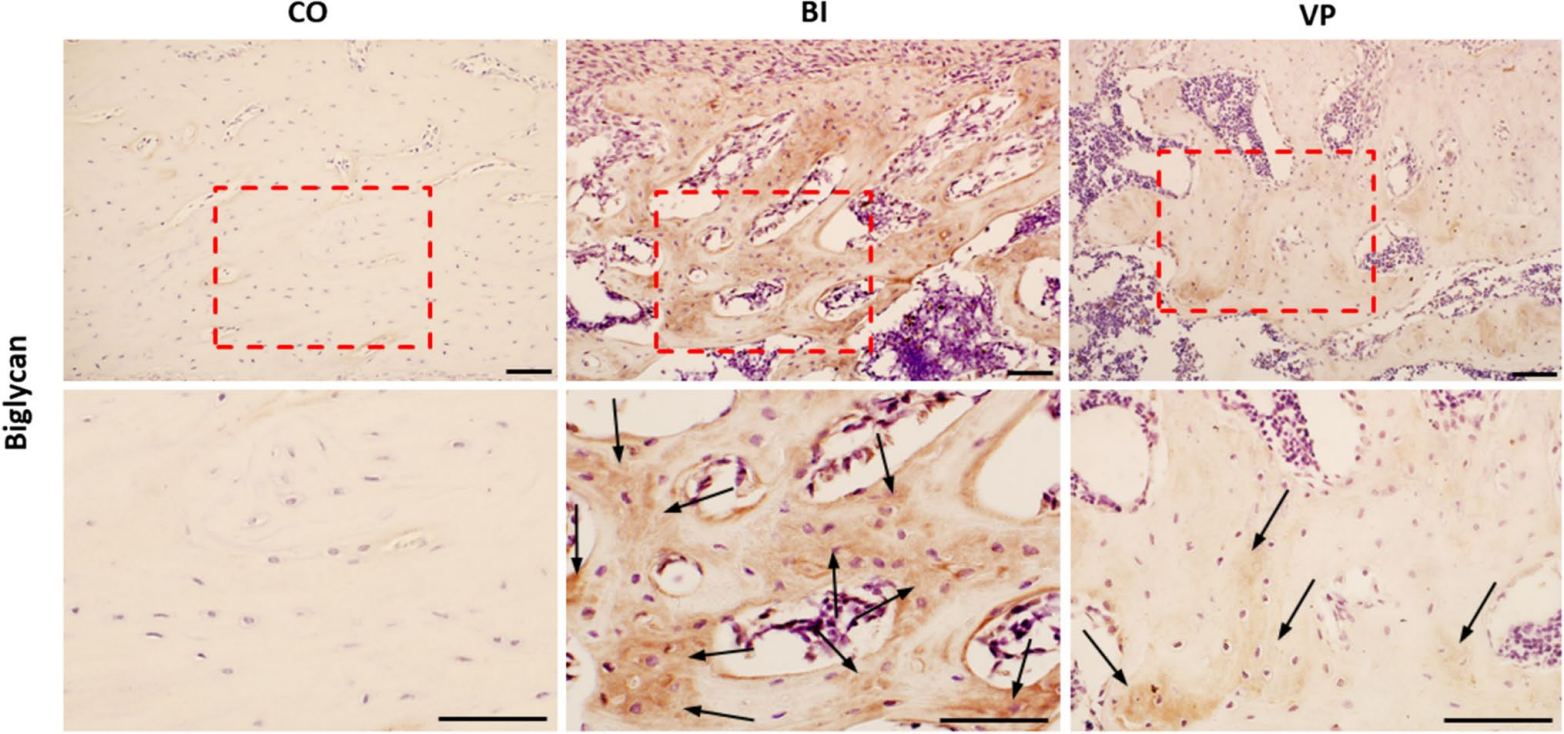
Representative images of biglycan immunostaining on day 21 after drill-hole injury. Top row: low magnification (×10). Bottom row: high magnification (×20). Black arrows indicate biglycan-positive areas in the bone matrix. Scale bar = 100 µm. *CO* Control, *BI* Bone injury, *VP* Vector-potential

Biglycan, a key proteoglycan in the bone matrix [29], was immunohistochemically analyzed (Fig. 5). Positive biglycan staining was observed in the bone matrix of both the BI and VP groups. However, the intensity of biglycan staining in the VP group was markedly reduced compared to that in the BI group, closely resembling the pattern observed in the CO group.

### VP stimulation enhances the metabolism of new bone

**Fig. 6:**
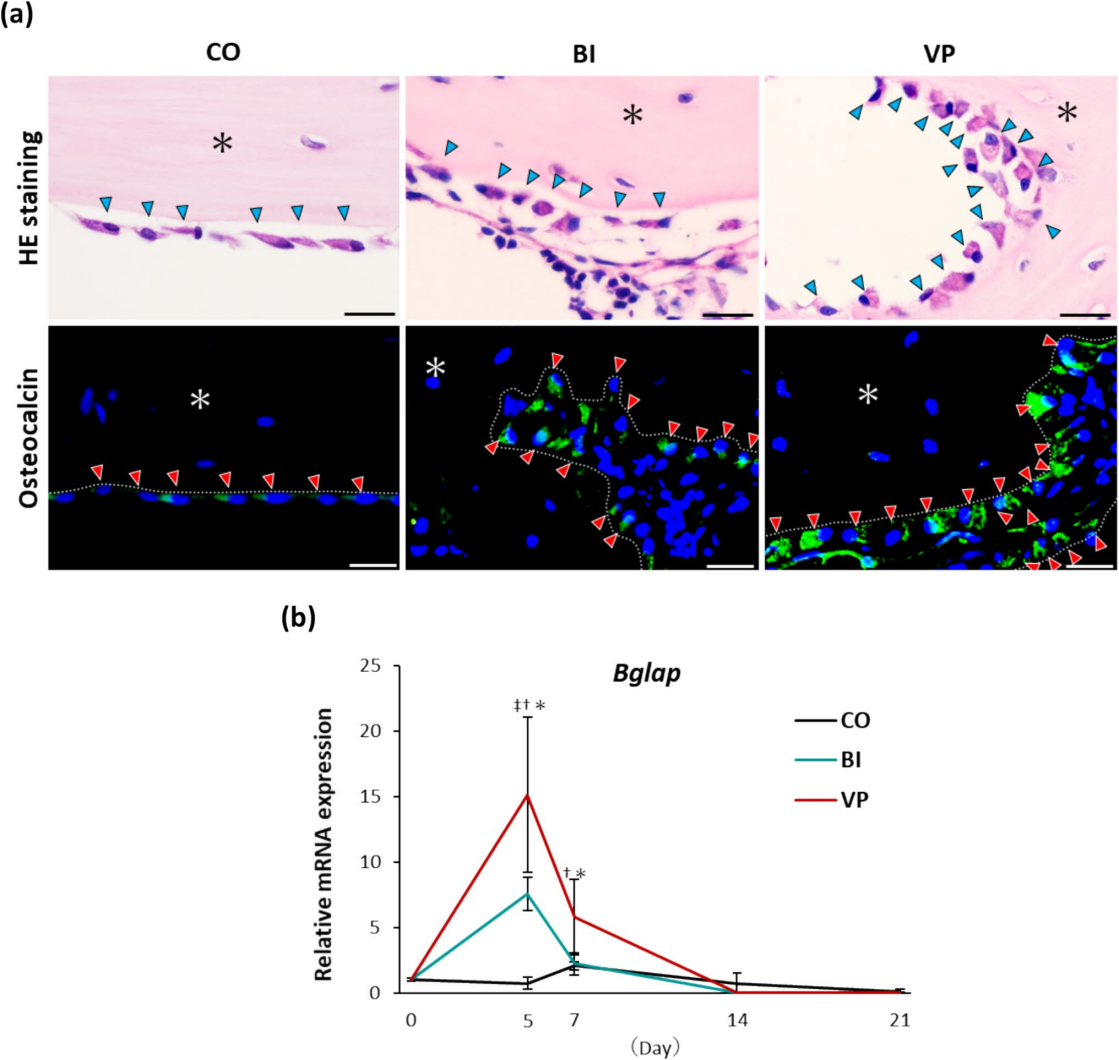
Bone formation in the drill-hole injury. **a** Representative images of HE staining (top) and Osteocalcin fluorescent immunostaining (bottom) on day 21 after bone injury. Arrowheads: Osteoblasts. *: Bone matrix. Dotted line: Bone surface. Scale bar = 20 µm. HE, hematoxylin/eosin. **b** Relative mRNA expression of *Bglap*. n = 3−6 per time point. ‡p < 0.05 (between CO and BI). †p < 0.05 (between CO and VP). *p < 0.05 (between BI and VP). *CO* Control, *BI* Bone injury, *VP* Vector-potential

HE staining revealed osteoblasts with more abundant cytoplasm in both the BI and VP groups than in the CO group. Notably, osteoblasts in the VP group exhibited a particularly prominent cytoplasm and a distinct cuboidal shape, in contrast to the flatter morphology observed in the BI group (Fig. 6a). Fluorescence immunostaining further demonstrated positive osteocalcin expression in the cytoplasm of osteoblasts in all the groups. However, osteocalcin expression was highest in the VP group, indicating enhanced osteoblastic activity (Fig. 6a). Gene expression analysis further supported these findings, showing significant upregulation of *Bglap* in response to bone injury in both the BI and VP groups. This upregulation was particularly pronounced in the VP group on days 5 and 7, with significantly higher expression levels than in the BI group (day 5: p = 0.0068; day 7: p = 0.0096; Fig. 6b).

**Fig. 7:**
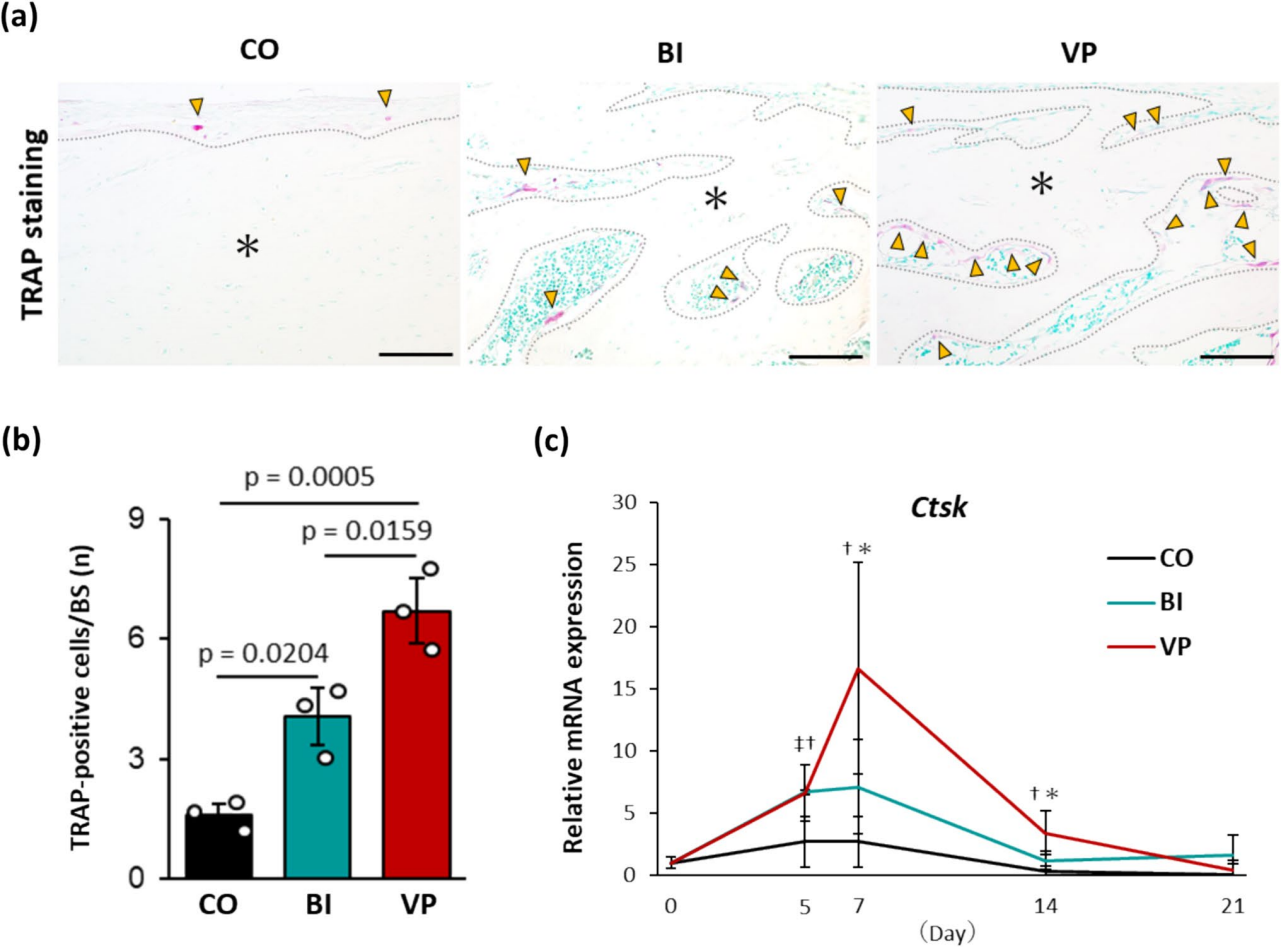
Bone resorption in the drill-hole injury. **a** Representative images of TRAP staining on day 21 after bone injury. Arrowheads: TRAP-positive cells. *: Bone matrix. Dotted line: Bone surface. Scale bar = 100 µm. TRAP, tartrate-resistant acid phosphatase. **b** Quantification of TRAP-positive cells. n = 3. **c** Relative mRNA expression of *Ctsk*. n = 3−6 per time point. ‡p < 0.05 (between CO and BI). †p < 0.05 (between CO and VP). *p < 0.05 (between BI and VP). *CO* Control, *BI* Bone injury, *VP* Vector-potential

To assess bone resorption, TRAP staining was performed on the surface of the new bone (Fig. 7a). TRAP-positive osteoclasts were significantly more abundant in the BI and VP groups than in the CO group. However, the VP group exhibited a significantly higher number of TRAP-positive osteoclasts than the BI group on day 21 (p = 0.0159; Fig. 7b). Further analysis revealed that the expression of *Ctsk*, which encodes cathepsin K, was upregulated early after bone injury in both the BI and VP groups. However, the expression levels were significantly higher in the VP group on days 7 and 14 (day 7: p = 0.0329; day 14: p = 0.0171; Fig. 7c).

## Discussion

To the best of our knowledge, this is the first study to investigate the effects of VP stimulation on bone healing. Histological analysis revealed that the VP group consistently exhibited significantly greater BV than the BI group at all evaluated time points. This was accompanied by increased new bone formation and enhanced bridging of the defect region. Notably, these differences were evident as early as day 7 post-injury, suggesting that VP stimulation promotes osteogenic activity in the early stages of bone healing and may facilitate early bone strength recovery.

However, bone strength is influenced not only by bone mass but also by bone quality, which encompasses mineral content and non-collagenous proteins [30]. Interestingly, toluidine blue staining revealed similar properties in the new bone of both the BI and VP groups on days 7 and 14. By day 21, however, the staining intensity was notably reduced in the VP group, resembling that of the CO group. This suggests more advanced mineralization in the VP group than in the BI group [31].

Toluidine blue staining induces metachromasia by reacting with proteoglycans containing sulfate groups, producing distinct staining patterns [32, 33]. Proteoglycans, such as biglycan, are abundant in the unmineralized bone matrix but decrease as mineralization progresses [34]. Consequently, as mineralization advances, the metachromatic reaction of toluidine blue diminishes [35, 36]. In the VP group, this reaction was weaker on day 21 than in the BI group, coinciding with a markedly weaker biglycan-positive reaction. These findings suggest that calcification progressed more rapidly in the VP group, likely due to an earlier reduction in biglycan, with this effect becoming more pronounced from at least day 21 onwards. In fact, while differences in BMD between the VP and BI groups were not significant until day 14, the VP group exhibited a significantly higher BMD by day 21. Although mechanical analysis was not performed in this study, our findings suggest that VP stimulation enhances both bone mass and quality, ultimately leading to early structural reinforcement of the drill-hole injury site.

Bone healing is a dynamic process that depends on continuous metabolism through the coordinated actions of osteoblasts and osteoclasts [37]. Osteoblasts, derived from mesenchymal stem cells, are responsible for synthesizing key bone matrix proteins, including osteocalcin, alkaline phosphatase, and type I collagen [38]. In contrast, osteoclasts, originating from hematopoietic stem cells, resorb bone by secreting proteolytic enzymes such as cathepsin K and matrix metalloproteinase 9, along with acids, to degrade the bone matrix [39]. The interaction between these cell types is essential for effective bone metabolism and healing.

In our study, osteoblasts in the VP group exhibited a cuboidal shape and abundant cytoplasm, indicative of high matrix-synthesis capacity, and were more prominent than those in the BI group [31, 40, 41]. Similarly, osteoblasts in the VP group showed a strong cytoplasmic positive reaction for osteocalcin, along with significantly upregulated *Bglap* expression on days 5 and 7 compared to the BI group. These findings suggest that VP stimulation enhances osteoblast activity, increases matrix-producing capacity, and promotes bone healing.

Furthermore, VP stimulation influences osteoclastic activity. The VP group showed a significantly higher number of TRAP-positive osteoclasts on day 21 and a significant upregulation of *Ctsk* expression on days 7 and 14 compared to the BI group. These findings indicate that VP stimulation promotes both bone formation and resorption, thereby enhancing the metabolism of new bone in the defect region.

A key finding of this study is that the bone metabolism-promoting effect of VP stimulation was evident from the early phase of healing following drill-hole injury. In the VP group, compared to the BI group, new bone formation at the defect region increased earlier, while immature bone within the intramedullary region decreased more rapidly, as confirmed by both µCT analysis and histological observations. Previous studies have reported that in the drill-hole injury model, the healing process is characterized by an initial transient formation of new bone within the intramedullary region, which is subsequently resorbed, followed by a transition to bone formation in the defect region [42–44]. Based on these findings, it is suggested that the transition from intramedullary-region bone formation to defect-region bone formation occurs earlier in the VP group. In other words, VP stimulation promotes bone metabolism from the early phase of healing, rather than only in the later stages, thereby accelerating the overall healing process following drill-hole injury compared to the BI group. These results suggest that early application of the VP transformer after fracture may enable minimally invasive and effective fracture healing from an earlier stage in the treatment process.

This study had certain limitations. First, we used a drilling-induced cortical bone injury model, which, while ensuring procedural simplicity and reproducibility, does not fully replicate typical fractures. Consequently, the true efficacy of VP stimulation in real-world fracture treatment remains to be established. Additionally, although this study primarily focused on bone formation and resorption to demonstrate the bone-healing effects of VP stimulation, the specific underlying mechanisms and pathways remain unclear. As fracture healing is a multi-stage biological process, bone formation and resorption are crucial, but they represent only one aspect of healing. Therefore, further research is needed to investigate the effects of VP stimulation on other critical factors, such as immune responses [45, 46], vascularization [47, 48], and mechanical stress [49, 50]. Furthermore, this study did not investigate the possible side effects associated with VP stimulation. While its non-contact nature may reduce the likelihood of burns, electric shocks, or allergic reactions, a thorough risk assessment is necessary to ensure both the safety and therapeutic reliability of this method in fracture treatment. Addressing these limitations in future research could provide even stronger support for the practical application of the VP transformer in bone fracture treatment.

In conclusion, our study demonstrates that non-contact electrical stimulation via a VP transformer enhances bone healing in drill-hole injury models. These findings highlight its potential as a novel, non-invasive therapeutic strategy for fracture management.

## Data Availability

Data supporting the findings of this study are available from the corresponding author upon request.
